# Development of Family Medicine training in Botswana: Views of key stakeholders in Ngamiland

**DOI:** 10.4102/phcfm.v7i1.865

**Published:** 2015-08-31

**Authors:** Radiance M. Ogundipe, Robert Mash

**Affiliations:** 1 Division of Family Medicine and Primary Care, Faculty of Medicine and Health Sciences, Stellenbosch University, Tygerberg, South Africa

## Abstract

**Background:**

Family Medicine training commenced in Botswana in 2011, and Maun was one of the two sites chosen as a training complex. If it is to be successful there has to be investment in the training programme by all stakeholders in healthcare delivery in the district.

**Aim:**

The aim of the study was to explore the attitudes of stakeholders to initiation of Family Medicine training and their perspectives on the future roles of family physicians in Ngami district, Botswana.

**Setting:**

Maun and the surrounding Ngami subdistrict of Botswana.

**Methods:**

Thirteen in-depth interviews were conducted with purposively selected key stakeholders in the district health services. Data were recorded, transcribed and analysed using the framework method.

**Results:**

Participants welcomed the development of Family Medicine training in Maun and expect that this will result in improved quality of primary care. Participants expect the registrars and family physicians to provide holistic health care that is of higher quality and expertise than currently experienced, relevant research into the health needs of the community, and reduced need for referrals. Inadequate personal welfare facilities, erratic ancillary support services and an inadequate complement of mentors and supervisors for the programme were some of the gaps and challenges highlighted by participants.

**Conclusion:**

Family Medicine training is welcomed by stakeholders in Ngamiland. With proper planning introduction of the family physician in the district is expected to result in improvement of primary care.

## Abstract (fr)

**Contexte:**

On a commencé la Formation en Médecine familiale au Botswana en 2011, et Maun était l’un des deux sites choisis comme centre de formation. Pour que le projet réussisse il faut que tous les acteurs des prestations de soins de santé de la région s’investissent dans le programme de formation.

**Objectif:**

L’étude avait pour but d’examiner les attitudes des parties prenantes envers le lancement d’une formation en médecine familiale et leurs points de vue concernant le futur rôle des médecins de famille dans le district de Ngami, au Botswana.

**Lieu:**

Maun et le sous-district avoisinant de Ngami au Botswana.

**Méthodes:**

On a effectué des entrevues avec des intervenants clé expressément sélectionnés parmi les services de santé du district. On a enregistré, transcrit et analysé les données suivant la méthodologie du cadre.

**Résultats:**

Les participants ont bien accueilli le développement d’une Formation en Médecine familiale à Maun et espèrent que cela amènera une amélioration de la qualité des soins primaires. Les participants désirent que les directeurs et les médecins de famille offrent des soins de santé holistiques et une expertise de meilleure qualité que celle fournie à l’heure actuelle, fassent des recherches appropriées sur les besoins en santé de la communauté, et réduisent le besoin de référence. Les participants ont souligné certaines lacunes et défis tels que les services insuffisants de bien-être personnel, les services auxiliaires de soutien irréguliers et une équipe insuffisante de mentors et de surveillants pour le programme.

**Conclusion:**

Les acteurs du Ngamiland ont bien accueilli la formation en médicine familiale. Avec une bonne planification, la mise en place d’un médecin de famille dans le district devrait améliorer les soins primaires.

## Introduction

In response to the Alma-Ata Declaration of 1978, many developing countries embraced primary health care (PHC) as the most cost-effective approach to providing accessible and affordable health care for their people.^1^ However, the benefits of this comprehensive and equitable approach to health care were not achieved in most countries of sub-Saharan Africa due to failure to address the socio-economic determinants of health problems, the introduction of vertical and disease-orientated health programmes in a selective PHC system, a short-term focus and a *laissez faire* approach to the emergence of unregulated commercialisation in health provision.^2,3^ The World Health Report 2008, however, reiterates the need to strengthen PHC as the cornerstone of health systems.^3^ The report recommends transformation from a focus on specific diseases and vertical programmes to PHC that comprehensively responds to all of the people's health needs. Furthermore, the report argues that countries with successful PHC systems have included doctors with postgraduate training in Family Medicine or general practice.^3^

Like many other African countries, Botswana adopted PHC as their strategy to improve healthcare delivery. To this end, the Government made organisational changes at both national and local levels. A department of Primary Health Care was established at national level to coordinate preventive, promotive, curative and rehabilitative health services. Prior to 2010 PHC was managed separately by the Ministry of Local Government, but it was subsequently transferred to the Ministry of Health.

District health teams were established to oversee implementation of the Government health policies at local level and to encourage community participation in healthcare delivery. The PHC approach in Botswana has been largely nurse-driven, with various different cadres of nurses providing the interventions. PHC is supported by a network of primary hospitals, which are currently functioning poorly with many gaps in service provision. A number of new and well-equipped district hospitals have been built, with posts for general specialists such as obstetricians, paediatricians and surgeons.

Botswana established its first medical school in 2008 and has just graduated its first fully home-trained medical graduates. There has been high attrition amongst the medical workforce over the years, with recruitment skewed in favour of secondary and tertiary care settings. In 2012 the density of doctors and nurses per 10 000 population was 3.4 and 31.4 respectively, and significantly lower in rural districts than in urban districts.^4^ This has made it difficult to achieve the goal of accessible, high-quality PHC services at community level.^4^ Access to specialist care is also difficult, and leads to significant mortality and complications as a result of referring patients over long distances.^4^ Many of the medical officers in the district are currently foreign graduates with little understanding of local language and culture, a wide range of competencies, and no postgraduate education in Family Medicine.

In the light of these shortcomings in healthcare provision, Botswana has realised that introducing family physicians as expert generalists into its district healthcare system may help to solve some of these problems. Family Medicine was one of the initial postgraduate programmes introduced at the School of Medicine at the University of Botswana, and two training complexes were created in Maun and Mahalapye. The Botswana Health Professions Council has created a register for family physicians and the Ministry of Health appears committed to creating posts for the first cohort of locally trained family physicians in 2016. Recent policy documents suggest that family physicians will initially be placed at primary hospitals with outreach to the PHC platform.^4^

Although the core values and principles of Family Medicine are shared globally, the specific competencies and organisational principles that define the role of Family Medicine need to be explored in each region where the specialty is introduced.^5^ The training programme for family physicians in Botswana has been significantly influenced by the established programme in South Africa.^6^ The Botswana family physician is expected to have competencies appropriate to the District Health Services in the following clinical areas: Cardiology, Neurology, Nephrology, Gastro-enterology, Haematology, Oncology, Respiratory medicine, Endocrinology and Musculoskeletal, Dermatology, Ophthalmology and Ear-Nose-Throat (ENT); Consultation Skills; Women's Health; Internal Medicine; Surgery; Infectious diseases, HIV and/or AIDS, Tuberculosis (TB) and sexually transmitted diseases, and Emergency Medicine.^7^

It is expected that the family physician in Botswana will play the following roles in the health system:

- Practitioners with thorough knowledge, professionalism and requisite skills in Family Medicine, particularly those critical to the local environment;
- communicators and collaborators;
- managers capable of providing evidence-based, cost-effective patient care;
- advocates able to identify important social determinants of health affecting their patients and to advocate for their resolution;
- scholars committed to lifelong learning and clinical governance, and
- teachers educating and mentoring students and junior colleagues in the discipline of Family Medicine.^7^

These roles for the family physician in the district need to be explored with all stakeholders, taking into cognisance the district health needs, availability of other specialists, and cultural and socio-economic factors affecting the community.^8^

This study therefore sought to explore the perspective of key stakeholders in Maun and the surrounding Ngami subdistrict of Botswana on the new Family Medicine training programme and the future role of the family physician in the district health system.

## Research methods and design

### Study design

The study design was qualitative, utilising in-depth recorded interviews with each of the relevant stakeholders.

### Setting

Ngami subdistrict is the southern half of the North West District of Botswana. The entire district, also known as Ngamiland, is made up of two subdistricts: Ngami and Okavango. The entire Ngamiland has a population estimated at 133 000.^9^ Ngami subdistrict has a population estimated at 56 865,^9^ and consists of Maun, a rural town with a population estimated at 48 000. It is divided into eight wards, each with a clinic or health post. There are 23 surrounding villages with a total estimated population of 8865.^9^

Health care in Ngami subdistrict is coordinated by a District Health Management Team consisting of the hospital arm and the PHC arm. Ngami subdistrict is served by 68 mobile clinic stops, 17 health posts, and five clinics with maternity units, three other clinics, and a district hospital. The district hospital, Letsholathebe II Memorial Hospital, is located in Maun and serves as a referral centre for all the facilities in Ngamiland. Although called a district hospital, it has separate specialist departments such as Medicine, Surgery, Obstetrics and gynaecology and Paediatrics.

The PHC clinics are run by PHC doctors, without postgraduate training, who consult patients, carry out minor procedures like suturing of lacerations, and refer patients that need admission. There is usually one doctor assigned to a clinic as well as general or family nurse practitioners, who also consult. The doctors in charge of the clinics regularly consult on a weekly outreach basis at other health facilities in the district to conduct health programmes on issues such as antiretroviral therapy or TB clinics. The nurses rely on the doctors for guidance whenever they are available at their clinic, and consult with them when they have patients with conditions beyond their capability. Although usually administered by the nurses, the district health programmes are also supervised and at times run by the clinic doctors.

The Family Medicine training programme in Botswana seeks to provide family physicians with enhanced competence and professionalism to support and strengthen healthcare delivery at the primary care clinics and primary hospitals. The training is a four-year programme entailing core modules and clinical rotations. The core modules include Communication, Ethics and Professionalism; Clinical research and Medical Literature; Principles and Techniques of Medical Education; Public Health Principles and International Health; Human Growth, Development and Family-orientated Primary Care; and Health Care Management and Administration. The clinical rotations are in the disciplines of Internal Medicine; Cardiology; Nephrology; Neurology; Endocrinology; Haematology; Musculoskeletal and Respiratory Medicine; Oncology; Women's Health; Infectious diseases – HIV and/or AIDS, TB, STDs; Surgery; Emergency Medicine; Dermatology; ENT; and Ophthalmology.^7^

During the rotations the registrars’ learning is supervised by consultants at the district hospitals to which they are attached as well as family physicians who oversee their learning outcomes. The family physicians are responsible for supervising the primary care exposure of the registrars to ensure their grasp of the essential concepts of Family Medicine. There are eight registrars in the programme presently. At the end of the clinical rotations registrars are assessed for their ability to apply clinical knowledge, communication and clinical skills, judgement and decisiveness, and professional attributes and values.

### Selection of key stakeholders

Purposeful sampling was used to select the interviewees who were key managers within the local district health system, and who had control over the training environment and service delivery platform, as well as senior clinicians. Fifteen key stakeholders were defined as follows: the coordinator of the district health management team, the managers of the district hospital (three people), the managers of PHC (three people), a principal medical officer from the hospital and PHC (two people), three doctors in charge of clinics, and three nurses in charge of clinics.

### Data collection

Interviews were conducted over a six-week period in 2012, lasted between 30 and 45 minutes each, and were conducted in English by the researcher at a place convenient for the interviewees. An interview guide, shown in Box 1, was used to conduct the in-depth interviews, but respondents were allowed to express themselves freely in answering.

**BOX 1:** Interview guide.

Opening question: ‘How do you feel about having these new Family Medicine registrars in the district?’
Further questions were then used to explore the respondent’s attitude towards the development of a Family Medicine training complex: How important is the development of the Family Medicine training complex for you? What do you think are some of the benefits of developing a Family Medicine training complex? What do you look forward to as the Family Medicine training complex develops? What are some of the difficulties that you anticipate in the development of a Family Medicine training complex? Do you have any concerns regarding the development of a Family Medicine training complex?
These questions were used to explore their perspective on the future role of family physicians and Family Medicine registrars in the district health care delivery system: Once these registrars graduate and become specialist family physicians, what role do you see them fulfilling in the district health system? What concerns do you have about having these specialist family physicians in the future? Do you anticipate any other difficulties? What do you think are the pros and cons of having specialist family physicians in the district health system? What impact or effect do you think these specialist family physicians will have on the district health system?
These questions were used to identify gaps in the training opportunities and facilities that need to be addressed to meet the standards of Family Medicine in sub-Saharan Africa: What changes do you think are necessary to your facility to make it suitable for training of Family Medicine? What are the main training opportunities that your facility can offer to registrars in Family Medicine? What essential training opportunities do you think the district will struggle to provide? Do you anticipate any issues with the supervision of these registrars?

HCW, healthcare workers.

The opening question was: ‘How do you feel about having these new Family Medicine registrars in the district?’ Subsequent questions explored attitudes towards the training programme, perspectives on the future roles of family physicians, and issues that needed to be addressed by the health services. The interviews were audio-recorded, backed up by field notes, transcribed verbatim by a research assistant and checked in comparison to the recording by the researcher.

### Data analysis

The transcribed data were analysed using the framework method.^10^ After thorough familiarisation with the data a thematic index was developed by coding the data and organising the codes into categories using the Atlas-ti qualitative analysis software. The transcripts were indexed by systematically applying the codes in the thematic index to all the data. Charting was done to bring all data with the same codes together. These were then interpreted by the researcher to identify the range and depth of different themes and any relationships between themes.

### Ethical considerations

The research was approved by the Health Research Ethics Committee at Stellenbosch University (Reference Number N/11/06/187) and the Ministry of Health Botswana Research and Ethics Board. The study was conducted according to the ethical guidelines and principles of the International Declaration of Helsinki, South African Guidelines for Good Clinical Practice and the Medical Research Council Ethical Guidelines for Research.

## Results

Thirteen interviews were conducted, as the head of the district health management team had resigned and one principal medical officer refused consent. Interviews were conducted with the district chief public health officer, two senior managers at the hospital, the principal medical officer in charge of the clinics, doctors in charge of four clinics, and nurses in charge of five clinics. One audiotape of an interview with a doctor in charge of a clinic was discarded due to poor quality. The key themes that emerged from the interviews are described below.

### Advent of Family Medicine training complex welcome

Most respondents were excited about the development of the Family Medicine training complex:

‘The coming of the Family Medicine training especially in Maun is really welcomed. It has offered to us as a nation, a background where we can have the knowledge and skills. It has brought to us a forum to discuss from time to time as doctors and be closely guided.’ (Doctor in charge of a clinic in Maun)

The training was expected to increase knowledge and information sharing through interactions between the registrars and other healthcare workers in the district. This was expected to facilitate upgrading of their skills and performance with improved health delivery to the community:

‘It is important. Like I said, it is going to benefit the community and the health workers, not only the community but also health workers. They can get more information and new ideas from doctors who are doing Family Medicine.’ (Nurse in charge of a clinic in Maun)

Some respondents felt the training would lead to better interaction between healthcare workers and the community, encouraging better understanding of community health needs whilst facilitating the appreciation of health services provided to the community. Others felt that the training will attract and enhance the retention of Batswana medical doctors who would have better understanding of the local culture and no language barrier. This, they felt, would improve patient-doctor interaction, and lead to better diagnosis and more holistic health provision at individual, family and community levels. Some felt that developing Maun into a Family Medicine training complex would offer the registrars an opportunity to experience life in a remote rural setting and encourage them to remain after graduation:

‘By training here also, they will know it's a nice place and whenever they are posted here they will not hesitate to come. I think it's a good thing.’ (Senior district administrator)

The Family Medicine training complex was seen as reducing inequity and bringing significant benefit to the community because of the remote location of Maun, far away from other urban centres where most of the specialists are usually based:

‘So, bringing the training this side is a good thing because it will help us to develop this area at this level of health care and training people this side directly will also increase the number of healthcare providers that you can have who can work directly here.’ (Principal medical officer)

**FIGURE 1:**
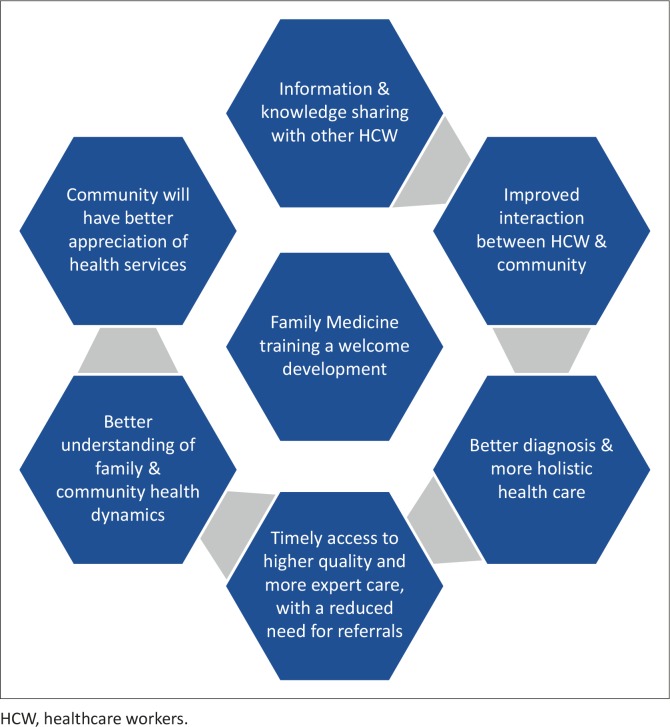
Expected benefits from the advent of Family Medicine training in Maun.

Other benefits expressed as expectations of Family Medicine training in Maun were improved economic and social growth. The perceived benefits of the training complex are summarised in Figure 1.

### Roles and expectations of Family Medicine registrars and family physicians

Family Medicine was expected to foster community engagement and champion community-oriented primary care. It was suggested that exposure of Family Medicine registrars and family physicians to the community would lead to a community diagnosis, with a better understanding of the causes of diseases and health conditions. Community identification with, acceptance of and participation in healthcare provision was also expected to result in greater benefits from the health services for the community:

‘I think they will be better prepared in the sense that they will be managing the cases and the conditions of the people who surround them. So, I see them as having been better prepared unlike studying in a different place and sending them to another place to go and serve, [*then you*] meet more challenges unlike when you started, knowing exactly the type of community you are serving.’ (Senior district nursing administrator)

Added one principal medical officer: ‘The most important activity to embark on is to understand exactly the community and how they function; what are their needs?’

The Family Medicine registrars and physicians were also expected to understand family dynamics and their role in the disease processes, which should lead to more holistic patient care.

Other roles expected of the registrars and family physicians as a result of their closer interaction with the community included primary care research into the health needs and burden of disease in the community, stated by a nurse in charge of a clinic in Maun as follows: ‘Family medicine being a specialty course on its own will enable further research and contribution into [*solving*] the problems patients are bringing forth.’

This primary care research, arising from issues identified within the community or at the primary care facility, was expected to yield findings that would be more acceptable to the community and to bring about more effective interventions.

Another role expected of the registrars and family physicians was provision of high-quality health care and mentoring of other healthcare providers. The family physicians are expected to provide guidance and avail current evidence-based ways of caring for patients. Respondents felt that the family physicians will provide a critical mass of skilled and capable practitioners that will support high-quality healthcare at the community level:

‘With this development, we can have people at hand and with proper organisation of specialist clinics regarding to health care in this district, we shall be able to have quite a number of skilled practitioners.’ (Doctor in charge of a clinic in Maun)

Timely access to expert generalist care and reduced referrals to other specialists was another benefit expected from the presence of the registrars and family physicians. Expert generalist care of commonly presenting and locally prevalent conditions across many fields of health care was expected to be provided by the family physicians, although this was conceptualised as a combination of skills from other specialties:

‘Family Medicine, as it incorporates all those specialities: obstetrics, gynaecology and what … I think the whole community will benefit because, now it will be a doctor who has done Family Medicine who will be able to do all those … in one person. So, you will be looking at the client holistically.’ (Nurse in charge of a peripheral clinic)

Development of other healthcare workers through information sharing and clinical mentoring was another role expected of the registrars and family physicians, as stated by this nurse in charge of a clinic in Maun: ‘So, they give information to staff members. So, the staff members benefit from the new information which they didn’t know. Thus, those working are being empowered.’

However, one participant felt the advent of Family Medicine registrars and family physicians was a threat to other medical practitioners. He explained further that this threat to the job security of other generalist medical officers could materialise after some years:

‘Those who are trained in family medicine, for them to work, they have to replace the doctors who were not trained then, that's … that's where I can see some kind of friction coming there because it means, getting jobs, losing your job to somebody else.’ (Doctor in charge of a clinic in Maun)

**FIGURE 2:**
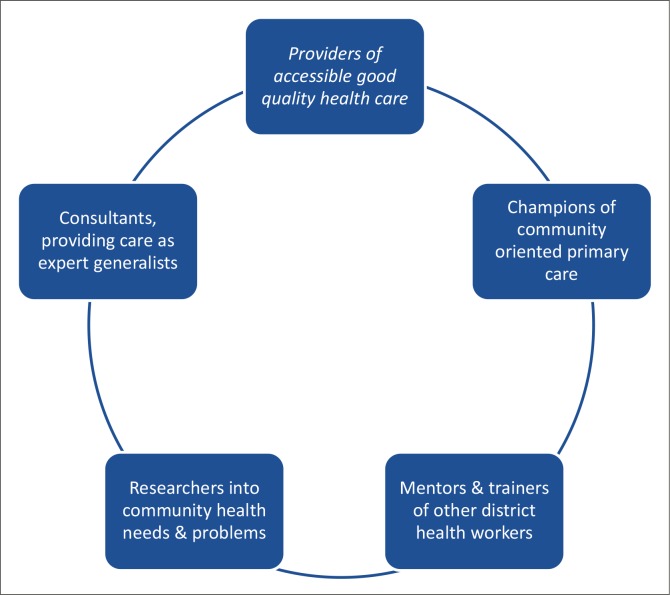
Summary of the roles expected from family physicians in Maun.

Participants did not anticipate problems with integration of the Family Medicine specialty into the district health system. Their views on the different roles of the registrars and family physicians are summarised in Figure 2.

### Gaps in training opportunities and challenges of developing Family Medicine training

A number of personal lifestyle challenges and expectations of the registrars in this rural area were discussed. Personal issues raised by participants included concerns about accommodation, transport, electricity, potable safe water and access to good-quality food. Although most respondents agreed that there is a need for the registrars to be trained at community level, they anticipated that this would be a challenge in terms of these personal issues:

‘They can, they have to be accommodated so we have to find a place for that in Maun. I mean if this accommodation can be found and then when you go to places where water … running water is the problem, or electricity is not provided.’ (Doctor in charge of a clinic in Maun)

Transport to ‘hard to reach’ areas such as the communities in the swamps of the Okavango Delta was viewed as particularly challenging. One participant actually felt that these personal issues may hinder recruitment for the programme, but others suggested starting with an initial low number of registrars and providing a retention allowance to compensate for the personal challenges involved in compulsory short- term rotations to rural facilities:

‘But I feel if we have to retain them in this area, we need to change the issues around accommodation and to place a specific retention allowance for such people who accept to do service to people in the remote [*areas*].’ (Senior health administrator in Maun)

Participants were concerned about the scale of the training programme and whether there would be sufficient infrastructure to accommodate a large number of registrars, even in the town of Maun. However, the general consensus was that the anticipated personal lifestyle challenges could be overcome by proper planning and resource allocation.

The general staff complement in the district health team was considered inadequate to provide a conducive learning environment to support the training of family physicians in Maun:

‘If you place a family physician in a facility that is understaffed, he might end up doing a job of a simple nurse who has just graduated and therefore dilute the whole reason why we place a family physician there.’ (Senior district health administrator)

They also expressed concern that there may not be an adequate complement of family physicians and other specialists to supervise and mentor the registrars and ensure good-quality professional exposure and training in Maun:

‘I think for a good training in Family Medicine, we would have loved to have all these specialists well represented here so that when the new physician is graduating, he or she becomes a rounded up somebody who has a hint of everything up to scratch.’ (Senior district health administrator)

The reason for the dearth of specialists was attributed to the remoteness of Maun, and there were suggestions that the Government should consider providing incentives to attract specialists to assist with the training. Participants also felt that there could be financial challenges to the development of Maun into a Family Medicine training complex. They were concerned that the present Government budgetary constraints may impact adversely on the training:

‘We need a budget which has been allocated to do those kinds of things to train the doctors. … even the people who train, maybe those professionals, who come from outside; the Government has to look into how the, the package … the remuneration for those kind of people, maybe it can be a challenge, a problem for the training to go on.’ (Nurse in charge of a peripheral clinic)

One participant felt that the Government ought to see the financial commitment to the training as an investment which would have considerable future benefits. Another felt that if the Government prioritised the training, it would be feasible. A further participant believed that coping with deficiencies and learning to improvise were characteristics of working in remote areas, and should be an essential aspect of the training:

‘I believe that is part of the training, to be able to work in any type of environment even if it is under a tent.’ (Doctor in charge of a clinic in Maun)

Another issue that participants felt could affect learning opportunities for the Family Medicine training in Maun was substandard ancillary facilities:

‘They will have loved to have all the right investigation support here, equipment like scan, sophisticated laboratories and X-ray facility. But what we have currently in Maun are just the basic things, and that might be a challenge to their learning process, it will limit them.’ (Senior district health administrator)

The inadequate support facilities were also perceived as a limitation to the effective performance of the family physicians after they qualify. Inadequate drug supply was also expressed as a concern that could limit learning opportunities for the registrars and affect their future role as family physicians in the district:

‘When they come here, do they have adequate drugs to use because that come from the central medical stores, and how regular[*ly*] are we going to receive our supplies?’ (Senior district nursing administrator)

### Supervision and mentoring of Family Medicine registrars

Participants felt that this was a crucial challenge to overcome for successful Family Medicine training. The need for adequate numbers of family physician trainers that have in-depth knowledge of Family Medicine and would be able to give the necessary hands-on training to the registrars was expressed:

‘We need people that are qualified enough to supervise and monitor the students. Students come to provide services but they need to be guided, oriented and monitored and supervised in what they are doing. So, it will take someone who is qualified to know the needs of the students of the Family Medicine programme to be able to offer the right supervision.’ (Principal medical officer in charge of clinics)

The need for specialists in other clinical specialties was also emphasised as a necessary prerequisite for the successful training of registrars, as the presently available family physicians will be inadequate to meet all aspects of their learning needs in other disciplines:

‘We need doctors, specialised doctors … who will be directly linked to the academics of these students, but who will be working in these hospitals where they will be attached to.’ (Senior district health administrator)

There was also concern about the lack of a local benchmark for the quality of the training programme, especially considering the need for adherence to international standards whilst training with a locally relevant curriculum:

‘Do we have the correct curriculum? That is going to determine the quality of doctors that we will, we are producing. … But if we don’t plan, again we don’t plan well in teaching of these students concerned, we will have so many graduates … but we will have mediocre type[*s*] of specialist[*s*].’ (Senior district health administrator)

The possibility that the textbooks recommended for training may not be locally relevant was expressed as a concern. It was suggested that having a formalised training institute for Family Medicine may be a better approach for planning and developing the training complex. It was also felt that the present clinics and hospital may be substandard in design and thus not meet the prerequisites for acceptable Family Medicine training:

‘The institutions in terms of infrastructure, for example, hospitals and clinics and other health facilities, were not constructed primarily for training. So they might not meet the prerequisites for training.’ (Principal medical officer in charge of clinics)

### Need for community awareness of Family Medicine

Some participants felt that a general lack of awareness of the nature and importance of Family Medicine by the community in Maun could be a challenge to the learning opportunities for the registrars and their future role as family physicians. The need to educate the community on the nature and importance of Family Medicine was seen as very important to the success of the training in Maun. It was considered that community education and mobilisation will help prepare the way for the training complex and facilitate the integration of the family physician into the district health system.

## Discussion

The participants welcomed the development of a Family Medicine training complex and anticipated better-quality and more holistic care, a reduced need for referral to higher levels of care, and more exploration of the social and family dynamics involved in health and disease. These expectations from key health stakeholders in the district suggest that they recognise many deficiencies in PHC and saw the advent of Family Medicine as part of a solution.

However, for respondents to see the introduction of Family Medicine training and later family physicians in the health system as a panacea for all the problems or deficiencies in primary care may also be unrealistic and create expectations that cannot be fulfilled. As the impact of Family Medicine was still largely aspirational, it was probably difficult for respondents to clearly identify the limits of the likely impact. Overall respondents were hopeful and expressed the need for the necessary budgetary allocation and incentives to ensure the successful development of the discipline and its integration into the health system of the country.

In a recent study exploring the views of key academic and government leaders in some African countries on Family Medicine, the respondents similarly expressed their expectation that Family Medicine will lead to improved quality and more comprehensive health care, and reduce referrals to the overburdened higher level of health care.^11^ This expectation was reported to be a reality in the Western Cape, South Africa, when the impact of Family Medicine on district healthcare delivery was evaluated.^12^

In another evaluation, also in the Western Cape, family physicians and registrars were perceived by the district health managers to have made positive impacts on the clinical processes of key medical conditions like trauma, HIV and/or AIDS care, TB and chronic non-communicable diseases.^13^ However, this impact was not uniform across the province, depending on the number of family physicians available and their differing abilities to function optimally. Although the impact was perceived to be in the early stages of development, participants in the study remarked that this positive impact had increased the acceptance of the specialty and the demand by junior doctors for training as family physicians. Other aspects of the district health system perceived to have been impacted positively by the family physicians and registrars included access to health care, coordination of health services, comprehensiveness of health service provision and efficiency of health delivery.^13^

The roles of family physicians in South Africa have been described as care provider, consultant to the PHC team, capacity builder and mentor to other healthcare workers; supervisors of junior doctors and medical students, and champion of clinical governance and community-orientated primary care.^12^ These were similar to the roles expected of family physicians and registrars by key health stakeholders in Maun, although they expressed a greater interest in research at community level. The roles identified in the study are also consistent with those agreed to in the regional consensus statement on Family Medicine.^8^

Challenges to the development of Maun as a Family Medicine training complex expressed by the stakeholders are indeed genuine concerns. The performance of the first cohort of family physicians trained will influence others who may consider training in the future. It will also influence the credibility and recognition of the discipline by specialists in other clinical domains.^12,13^

Inadequacies of training facilities, mentors and supervisors can be detrimental to the quality of the family physicians produced. Training programmes also tend to undergo a development journey, as has been seen in South Africa. Initial training was strongly based in the regional hospitals under the supervision of other specialists. This was because there were insufficient family physicians in the district platform and facilities were poorly developed to train on all the skills required. This initial model was also necessary to get buy-in from the other specialists on the training of family physicians. However, registrars often felt orphaned in specialist departments and were not trained in the correct context. Subsequently, however, training has largely shifted into the district under the direct supervision of family physicians.^12^ One can anticipate a similar shift over time in the Botswana context.

Poor working conditions may also discourage further intake of registrars into the training programme. A suggestion to donor organisations to commit 15% of their budgets to the strengthening of PHC systems in developing countries rather than vertical disease-orientated programmes may be an avenue to explore for financial leverage to address the working conditions and some of the infrastructural inadequacies that will be a challenge to the success of the programme.^14^

At the recent First Botswana Family Medicine Conference, held in May 2013 in Gaborone, the collaborative support of Stellenbosch University for the University of Botswana in the development of Family Medicine was acknowledged. This collaboration may help to build capacity amongst the new academic staff and faculty members.

There is a need in Botswana for family physicians to be employed in the health services as role models and mentors and not just as academics. There is also a need to decide whether they will be employed at the primary care level or at primary hospitals. If, as seems likely, they will initially be employed at primary hospitals to meet a critical skills gap, this will follow the pattern seen in South Africa, but differ considerably from the role of the frontline general practitioner in Europe and America. In South Africa the family physician as a well-trained scarce resource is now focusing more on the subdistrict as a whole, and whilst based at a hospital would provide extensive outreach to the primary care services.^15^ Botswana, with its relatively small population and middle-income status, seems more able to employ doctors at all the clinics, and could in future move towards ensuring they have postgraduate training. It was also proposed at the conference that the support of the private health sector should be explored in developing the Family Medicine programme.

Opinions of the key health stakeholders interviewed in this study seem strongly in favour of retaining Maun as a training location, rather than shifting the training closer to Gaborone, as suggested by some participants at the conference. This is supported by findings in the Western Cape and Gelukspan health ward in South Africa, where training of the registrars in rural communities enhanced their understanding of the health issues and increased the chance that they would remain in those communities after graduation.^12,14,16^ Relocating the training closer to an urban setting may also further perpetuate inequitable distribution of healthcare provision.^17^

### Limitations

A few participants might have expressed themselves more fully if the interview had been conducted in Setswana. The researcher (R.M.O.) was a Family Medicine registrar at the time this study was done, and has a deep desire for the successful integration of the specialty into the health system in Botswana. Although he did his level best to remain neutral whilst conducting the interviews, this interest could have affected his perception of the views expressed by the respondents. R.M.O. has worked as a senior level officer in the health team in Maun for more than six years, in both the clinics and the hospital. Most of the respondents are known to him and some have worked with him. This fact may also have affected the way they expressed their views, despite assurances of confidentiality. The Principal Medical Officer who declined to participate in the study for undisclosed reasons could have provided an alternative viewpoint, but his reason for declining is not known. Similarly, the audio file of one of the clinic doctors that was discarded owing to poor audio quality could have provided additional information. However, by continuous familiarisation with the data during the interviewing phase, it was concluded that no new themes were emerging after the eleventh interview.

### Implications and recommendations

The following recommendations can be made on the basis of this study:

- There is an urgent need to improve working conditions and develop incentive packages to attract and retain health workers in the rural areas of Botswana.
- Maun should be retained as a rural training complex within the Family Medicine training programme.
- The University of Botswana will need to explore ways of incorporating family physicians in clinical practice into the programme.
- The supply of resources to district level medical facilities should be improved so as to create a better training environment.
- Maun as a training complex will need to develop measures for evaluating the impact of the registrars and Family Medicine on PHC in the district.
- Research to evaluate the cost-effectiveness and impact of the introduction of family physicians in similar districts in sub-Saharan Africa will be useful to demonstrate objectively the benefits of Family Medicine.

## Conclusion

Key health stakeholders in Ngamiland support the need for the training of Family Medicine registrars and placement of family physicians in the district health services in Maun. The roles described for the family physicians by the stakeholders are consistent with the roles identified in South Africa and the broader region.

Gaps in facility infrastructure, resources and learning opportunities may be a challenge to the development of the training complex.

There was a generally optimistic outlook by the stakeholders that with proper planning the challenges would be overcome, and PHC in the district would benefit from the introduction of Family Medicine registrars and family physicians.
